# Concentration‐Driven Li^+^ Solvation Engineering with TDMAP‐Based Porphyrin Additives for Dendrite‐Free Li Metal Batteries

**DOI:** 10.1002/advs.76009

**Published:** 2026-06-09

**Authors:** Pooria Afzali, Jian Wang, Sergio Rodriguez, Jin‐Hyun Chang, Luca Magagnin, Maximilian Fichtner

**Affiliations:** ^1^ Helmholtz Institute Ulm For Electrochemical Energy Storage (HIU) Ulm Germany; ^2^ Dipartimento Di Chimica, Materiali e Ingegneria Chimica “Giulio Natta” Politecnico di Milano Milan Italy; ^3^ Institute of Nanotechnology, Karlsruhe Institute of Technology (KIT) Karlsruhe Germany; ^4^ Department of Energy Conversion and Storage Technical University of Denmark Lyngby Denmark; ^5^ Ulm University Institute for Inorganic Chemistry II Ulm Germany

**Keywords:** electrolyte additive, Li metal batteries, Li^+^ solvation shell, porphyrin, SEI

## Abstract

The performance of lithium metal batteries is strongly influenced by Li^+^ solvation and solid electrolyte interphase (SEI), which governs dendrite growth, cycling stability, and Coulombic efficiency. Herein, to modulate Li^+^ solvation structure, the tetrakis(4‐N, N‐dimethylaminophenyl)porphyrin (TDMAP) is introduced as an additive in carbonate‐based electrolyte, further reinforcing SEI stability. Spectroscopical measurements reveal that TDMAP displaces ethylene carbonate (EC) molecules from the first solvation shell, partially forming mixed coordination environments and acting as a ligand that modulates the Li^+^ solvation shell and alters its interaction with PF_6_^−^ anions (Li^+^–NMe_2_–PF_6_^−^). Results show that the electrolyte with optimal 3 mg mL^−1^ TDMAP (TDMAP‐3) enables and promotes smooth, dense Li deposition and a LiF‐rich and nitrogen‐rich SEI on the condition of the expected Li^+^ solvation structure shell, in contrast to the dendritic and porous Li plating morphology as observed in the base electrolyte. Consequently, the cell with TDMAP‐3 additive reduces the nucleation overpotential from 62 to 20 mV, achieves high Coulombic efficiency (∼99%), and the Li–LiFePO_4_ full cell maintains capacity retention of 97.3% over 450 cycles, demonstrating that porphyrin additive effectively regulates Li^+^ solvation and SEI composition for high‐performance Li metal batteries.

## Graphical abstract

The electrolyte engineering of introducing tetrakis(4‐N, N‐dimethylaminophenyl)porphyrin (TDMAP) is designed to modulate Li^+^ solvation structure and solid electrolyte interphase, where the interaction with PF₆^−^ anions is altered (Li^+^–NMe_2_–PF₆^−^). Consequently, the cells with optimal additive concentration achieve high Coulombic efficiency (∼99%), and stabilize for over 450 cycles, demonstrating the advantages of porphyrin additive for high‐performance Li metal batteries.

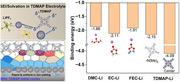

## 1 Introduction

The use of metallic lithium (Li^0^) as an anodic material in replacement of graphite is a key strategy to enhance energy density toward 500 Wh kg^−^^1^ [1, 2, 3]. However, the practical deployment of lithium metal batteries (LMBs), whether utilizing nickel cobalt manganese oxide (NCM) or lithium iron phosphate (LFP) cathodes, remains hindered by significant challenges [4]. The inherently low redox potential of lithium metal, while advantageous for increasing cell voltage, simultaneously induces severe instability at the interface [5, 6]. This instability, coupled with dendritic lithium growth, weak solid electrolyte interphases (SEIs), limited cycling stability, and sluggish interfacial kinetics, leads to rapid capacity degradation, safety concerns, and constrained charge–discharge rates [7, 8]. Therefore, developing effective approaches to stabilize lithium metal anodes (LMAs) against electrolyte decomposition and electrode‐electrolyte interfacial degradation is critical for advancing LMBs toward commercial viability.

As known, electrolyte additive engineering can serve as a powerful strategy to regulate interfacial chemistry and improve the stability of LMAs [9, 10, 11, 12]. Additives can operate through multiple mechanisms, including modulation of the SEI to form stable, ionically conductive, and mechanically robust layers that suppress parasitic reactions and dendrite growth [13, 14]. Additives may influence Li^+^ solvation structure, altering ionic transport pathways and enhancing deposition uniformity [15, 16, 17]. Such changes can increase the Li^+^ transference number by favoring cation transport and lowering the activation energy for ion migration through more favorable solvation or interfacial environments [18, 19]. Certain additives function as leveling agents, adsorbing onto protrusions and redistributing the local electric field to guide lithium deposition into lower potential or recessed regions, promoting smooth and dendrite‐free plating [20]. Others contribute to electrostatic shielding [21], mitigating high concentrations at nucleation sites. In addition, some molecules act as electron transfer mediators, enabling uniform charge flow at the interface, or as nucleation regulators, promoting dense and compact Li morphologies. These mechanisms often work synergistically to enhance ionic conductivity, suppress inhomogeneous Li growth, and improve Coulombic efficiency and cycling stability [22].

Among them, organic additives are widely explored in Li metal batteries due to their structural tunability and interfacial activity. They can adsorb on reactive sites, guide uniform Li^+^ deposition, and buffer volume changes during cycling [23, 24, 25, 26]. Porphyrins are heterocyclic π‐conjugated organic molecules composed of a planar aromatic macrocycle with a central N_4_‐core [27]. They exhibit extensive electron delocalization and reversible 16π, 18π, and 20π redox states, which enables ambipolar charge transport and stable multi‐electron processes [28, 29]. The macrocyclic structure also facilitates supramolecular interactions such as hydrogen bonding and π–π stacking, leading to porous and ordered assemblies [30]. Their chemical and electronic properties can be tuned either by modifying the peripheral positions of the ring (known as *meso‐substitution*) or by coordinating different metal ions at the center. These strategies allow precise control over the molecule's steric, electronic, and electrostatic features [31, 32]. Owing to these versatile characteristics, porphyrins are increasingly investigated for lithium metal batteries and other energy storage systems. For example, various porphyrin complexes have been studied as electrodes, such as [5,15‐bis(ethynyl)‐10,20‐bis(5‐methylthienyl) porphinato] copper (II) (CuDETMP) and [5,15‐bis(ethynyl)‐10,20‐bis(5‐chlorothienyl)porphinato]copper(II) (CuDETCP) in energy storage, where electron‐donating/withdrawing groups modulate redox potential and solubility. Chlorine substituents in CuDETCP interact with Li^+^ via p–π conjugation with thiophene, further enhancing capacity despite partial reversibility [33]. In aqueous zinc–organic batteries, tetraphenylporphyrin tetrasulfonic acid (TPPS) dissociates –SO_3_H groups to sulfonates that coordinate Zn^2^^+^, disrupt its solvation sheath, and guide uniform Zn deposition via Zn–porphyrin complexes; its macrocyclic cavity enables Zn^2^^+^/H^+^ synergistic storage [25]. In solid‐state lithium‐metal batteries, lithiated copper polyphthalocyanine (CuPcLi) in the electrolyte regulates Cu redox, accelerating fluorinated component decomposition to form LiF‐rich SEI, while Cu^2^^+^–O chemisorption and macrocyclic conjugation enhance Li^+^ release and transport [34]. Taking all into consideration, it remains unknown how the porphyrin works in the electrolyte and the related concentration effect on the Li^+^ solvation behaviors.

In this work, the tetrakis(4‐N,N‐dimethylaminophenyl)porphyrin (TDMAP) as a multifunctional electrolyte additive is initially proposed to modulate the solvation structure and stabilize Li metal anodes. Guided by its strong coordination capability and electron‐donating substituents, the concentration effects of TDMAP influencing Li^+^ solvation, SEI composition, and Li deposition behavior are comprehensively investigated, as revealed by spectroscopic analyses that TDMAP displaces ethylene carbonate (EC) molecules from the first solvation shell, forming mixed Li^+^–NMe_2_ and Li^+^–PF_6_^−^ coordination environments. The optimized concentration of TDMAP‐3 (3 mg mL^−^^1^) enables smooth, dense Li deposition with a LiF‐rich and nitrogen‐rich SEI. Electrochemically, with the use of the optimized TDMAP‐3 additives, Li‐Li cell reduces the nucleation potential from 62 mV (base electrolyte) down to 20 mV, the Li‐Cu cell achieves a Coulombic efficiency of ∼99%, and the Li‐LiFePO_4_ full cell maintains stable cycling over 450 cycles with the capacity retention of 97.3%. These findings highlight porphyrin‐based additives as a promising strategy regulate Li^+^ solvation and enable long‐lasting Li plating/stripping for high‐performance Li metal batteries.

## 2 Results and Discussion

**FIGURE 1:**
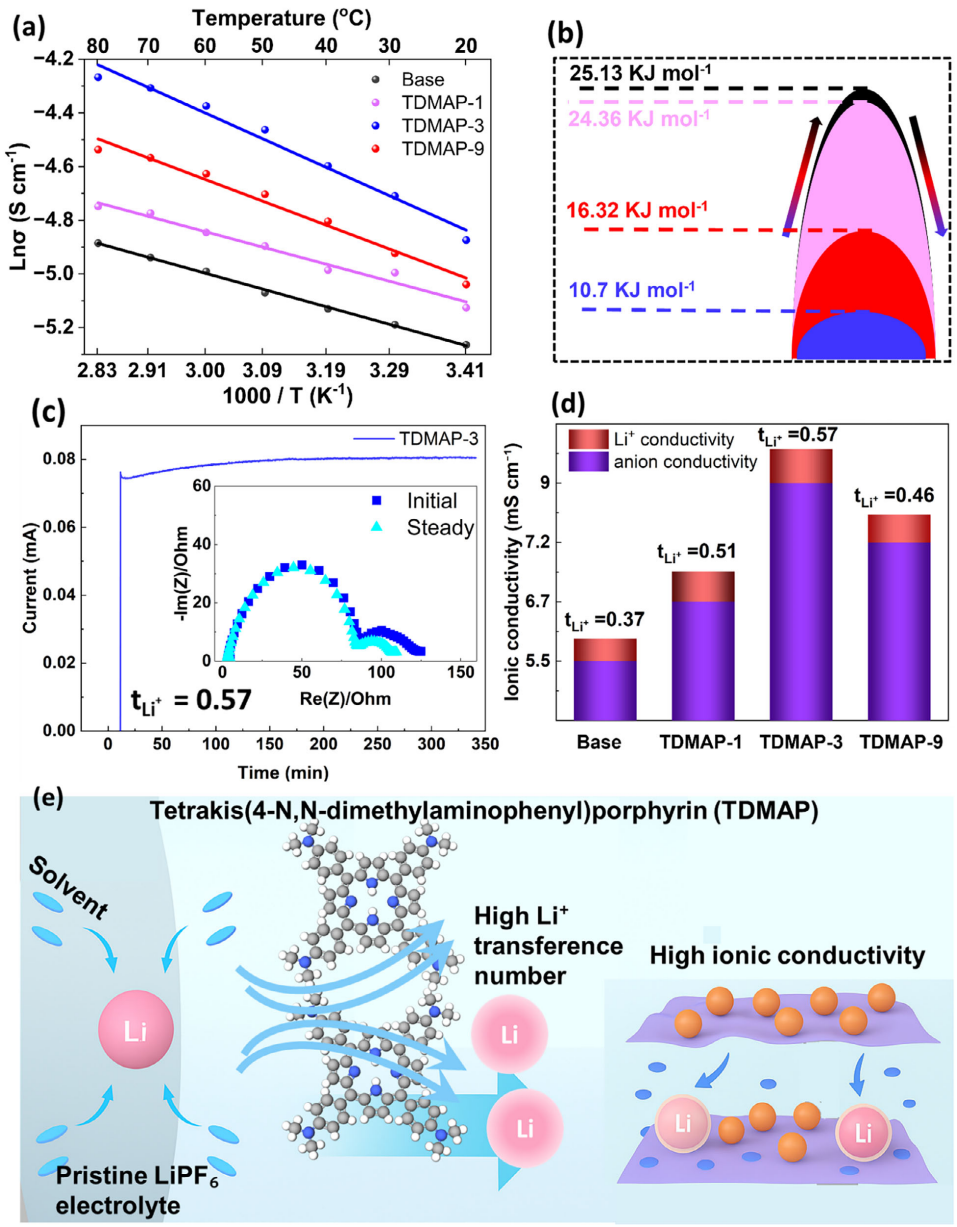
(a) Arrhenius plots of the ionic conductivities of base and TDMAPs electrolytes in temperature range of 20°C–80°C. (b) The activation energy (E_a_) of all electrolytes. (c) Li^+^ transference number measurement of TDMAP‐3. (d) Ionic conductivity and Li^+^ transfer number of different electrolytes systems. (e) Graphical illustration of enhanced ion migration in porphyrin‐containing electrolyte, demonstrating increased Li^+^ conductivity and mobility.

To investigate the electrochemical performance of TDMAP, a series of tests on the additive concentration was conducted in symmetric Li‐Li cells (Figure S1a). Among the key parameters for LMBs, ionic conductivity is firstly investigated. As shown in Figure 1a, all TDMAP‐containing electrolytes exhibited higher ionic conductivity than the base electrolyte over the full temperature range (20°C–80°C). This improvement can be attributed to the efficient ion transport provided by TDMAP through its interactions with dissociated Li^+^ ions. The porphyrin molecule, being electron‐rich, modifies the Li^+^ solvation shell and facilitates ion mobility. Correspondingly, the activation energy (E_a_) of TDMAP‐3, calculated using the Arrhenius equation, is 10.07 kJ mol^−^^1^, significantly lower than that of the base electrolyte (25.13 kJ mol^−^^1^) as shown in Figure 1b. The stability of the base electrolyte and the electrolyte containing TDMAP‐3 (Figure S1b) at room temperature was evaluated by using stainless disc as the working electrode. The results show that the onset oxidation potential of the TDMAP‐3 containing electrolyte is shifted to higher voltage compared to the base electrolyte, indicating an improved oxidative stability window due to the presence of the porphyrin additive. This enhancement can be attributed to the interaction between TDMAP and electrolyte components, which likely modifies the interfacial chemistry and delays electrolyte decomposition.

The lithium‐ion transference number (t_Li_^+^), determined by the Bruce–Vincent method, further confirms that the fast ionic transport channels introduced by the meso‐(4‐N,N‐dimethylaminophenyl) substituents at the electrolyte/electrode interface effectively lower the energy barrier for Li^+^ migration (Figure 1c,d). While TDMAP‐1 and TDMAP‐9 also outperform the base electrolyte, their performance does not match that of TDMAP‐3 (Figure S2). This could be attributed to insufficient additive concentration in TDMAP‐1, leading to a limited number of conductive pathways, or excessive additive in TDMAP‐9, causing aggregation at the interface. The optimized performance of TDMAP‐3 can be ascribed to π–π interactions and the electron‐rich bonds in the porphyrin structure, which improve Li^+^ transport efficiency [35, 36]. The conjugated π‐bonds within the TDMAP molecule enhance its dielectric response, promoting lithium salt dissociation and increasing Li^+^ mobility [37].

**FIGURE 2:**
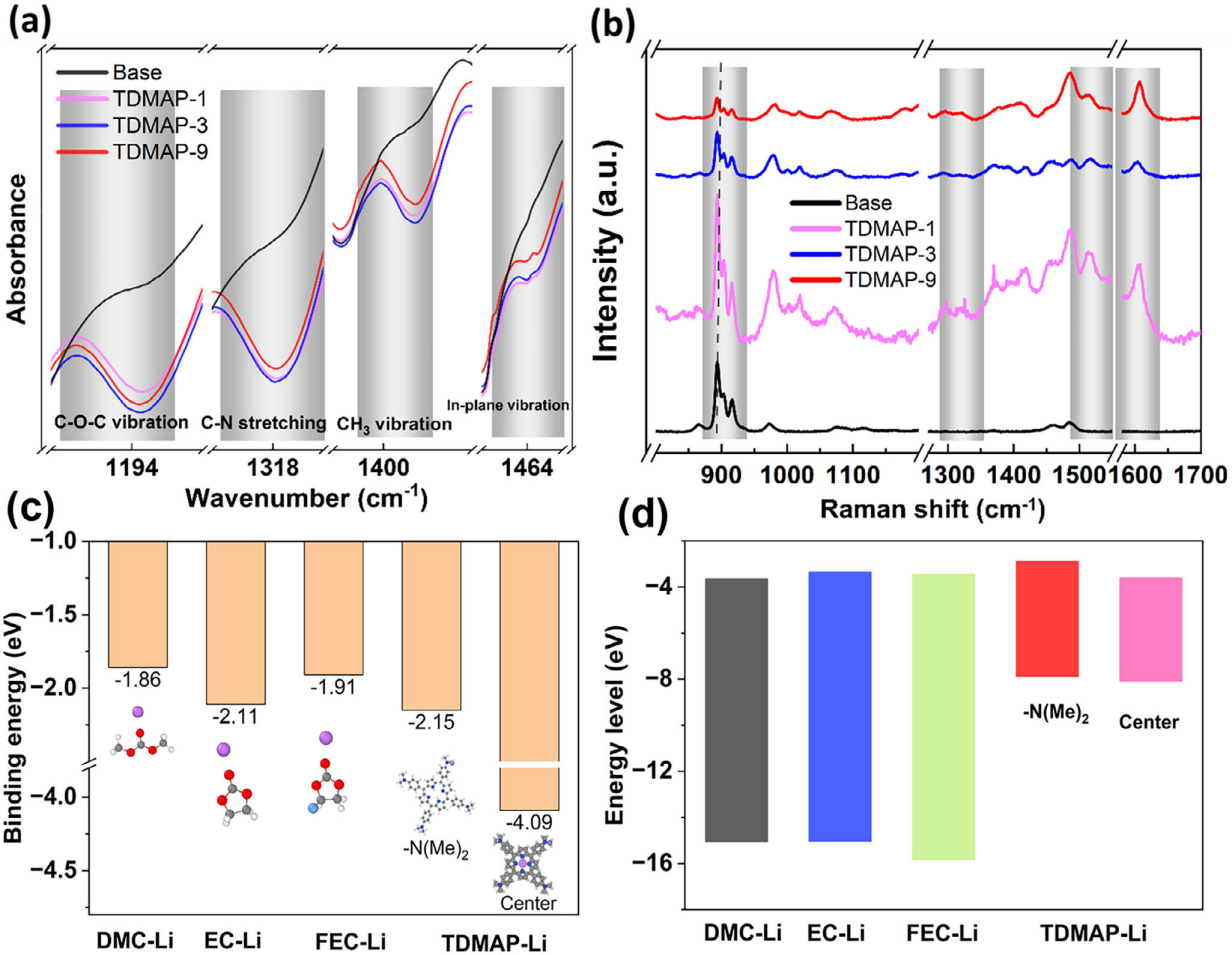
(a) Relevant magnified FT‐IR spectra of as‐prepared electrolytes at 1100−1500 cm^−1^. (b) Relevant magnified Raman spectra of as‐prepared electrolytes at 800–1700 cm^−1^ illustrating the shifts and molecular interactions. (c) DFT calculations of the binding energy of Li^+^ with solvents and TDMAP molecules. (d) HOMO‐LUMO energy levels for binding to Li.

The schematic illustration in Figure 1e shows that the TDMAP additive in the LiPF_6_ electrolyte, through its electron‐donating groups, modifies Li^+^ solvation and facilitates faster ion transport, thereby enhancing ionic conductivity. To explore the molecular interactions of TDMAP at different concentrations in the electrolyte, Fourier‐transform infrared (FTIR) spectroscopy and Raman spectroscopy were performed (Figure S3a,b). The FTIR spectra (Figure 2a) in the 1100–1500 cm^−^^1^ region reveal clear differences between the base electrolyte and the porphyrin‐containing systems (TDMAP‐1, TDMAP‐3, and TDMAP‐9). Upon adding TDMAP, new peaks emerge at ∼1194, 1318, 1400, and 1464 cm^−^^1^, which can be assigned to C─O─C and C─N stretching, CH_3_ bending, and in‐plane ring vibrations of the porphyrin macrocycle. The appearance and slight shifts of these peaks relative to the base electrolyte suggest that Li^+^ ions interact with the electron‐donating amino groups of the porphyrin. Additionally, a slight blue shift is observed in all spectra after adding the TDMAP. Raman spectroscopy results (Figure 2b) provide further insight. The ∼893 cm^−^^1^ band for TDMAP‐3 and TDMAP‐9 becomes less distinct and more broadened, indicating that fewer ethylene carbonate (EC) molecules directly coordinate with Li^+^. This is consistent with fewer EC molecules participating in the primary Li⁺ solvation shell. Moreover, all peaks corresponding to TDMAP‐1, TDMAP‐3, and TDMAP‐9 are shifted to lower wavenumbers (red‐shift) compared to the base electrolyte. This trend supports a concentration‐dependent softening of the EC ring‐breathing mode, consistent with progressive weakening of Li^+^–EC interactions as the porphyrin concentration increases. Mechanistically, this suggests that more EC molecules are displaced or perturbed from the Li^+^ first solvation shell, or that Li^+^ is increasingly present in ion‐paired or aggregated states, with the effect becoming stronger at higher porphyrin concentrations. The progressive red‐shift thus reflects the softening of the EC vibrational mode and a reduction in EC–Li^+^ coordination, consistent with the formation of alternative Li^+^ coordination environments, such as Li^+^–NMe_2_ interactions with TDMAP or Li^+^–PF_6_^−^ contact ion pairs [38]. In the 1284–1640 cm^−^^1^ region, bands at 1330–1350 cm^−^^1^ and ∼1480–1520 cm^−^^1^ arise from C─N stretching coupled with aromatic ring vibrations of the para‐N,N‐dimethylamino groups, indicating that their lone pairs interact with Li^+^ or the solvent and perturb the electron density on the porphyrin ring. This provides direct evidence that the C─N sites play a role in modifying the Li^+^ solvation shell, reducing EC's dominance and generating mixed coordination environments that can influence ion transport and SEI chemistry [39]. The ∼1600 cm^−^^1^ band, primarily corresponding to aromatic C═C stretching of the porphyrin macrocycle and phenyl rings (with some contribution from C─N coupling), shows a shift and sharpening that suggests coordination or charge transfer with Li^+^, as well as possible π–π stacking or ordering effects. Overall, these spectroscopic observations demonstrate that the porphyrin macrocycle and its dimethylamino substituents actively perturb the local electronic environment around Li^+^, and significantly modify and modulate its solvation structure, which may have direct implications for ionic transport and interphase formation [25].

The interactions between Li ion solvation and Porphyrin are demonstrated by the binding energies (BE) via density functional theory simulations. Among solvents (Figure 2c), EC exhibits the strongest interaction with Li, followed by fluoroethylene carbonate (FEC) and dimethyl carbonate (DMC). The results provide a clear thermodynamic and electronic basis for the observed behavior. The binding energies of the Li with the TDMAP is greater than that with any solvent molecule (−2.15 eV), indicating the preferential coordination of Li with the porphyrin's N(Me)_2_ group. This suggests that TDMAP can effectively compete with solvent molecules in the Li^+^ solvation shell. However, the drop on the highest occupied molecular orbital (HOMO) level from isolated to bind molecule indicates the stabilization of the complex (Figure 2d) [40]. This can be explained by the more localized nature of the HOMO in the carbonate solvents, which makes them more sensitive to Li binding, whereas the HOMO is more delocalized over the larger molecular structure in TDMAP. The lowest energy molecular orbital (LUMO) energies of the Li‐coordinated complexes provide insights into reduction behavior. The carbonate solvents exhibit lower (more negative) energies compared to TDMAP–Li, indicating a higher tendency to undergo reduction. In contrast, the higher LUMO energy of the TDMAP–Li complex suggests enhanced resistance to reduction, which may contribute to improved interfacial stability. The lower value for the TDMAP LUMO level indicates its preference to be reduced at the anode surface and modify the SEI composition, thereby decoupling solvation structure regulation from interfacial decomposition [41].

**FIGURE 3:**
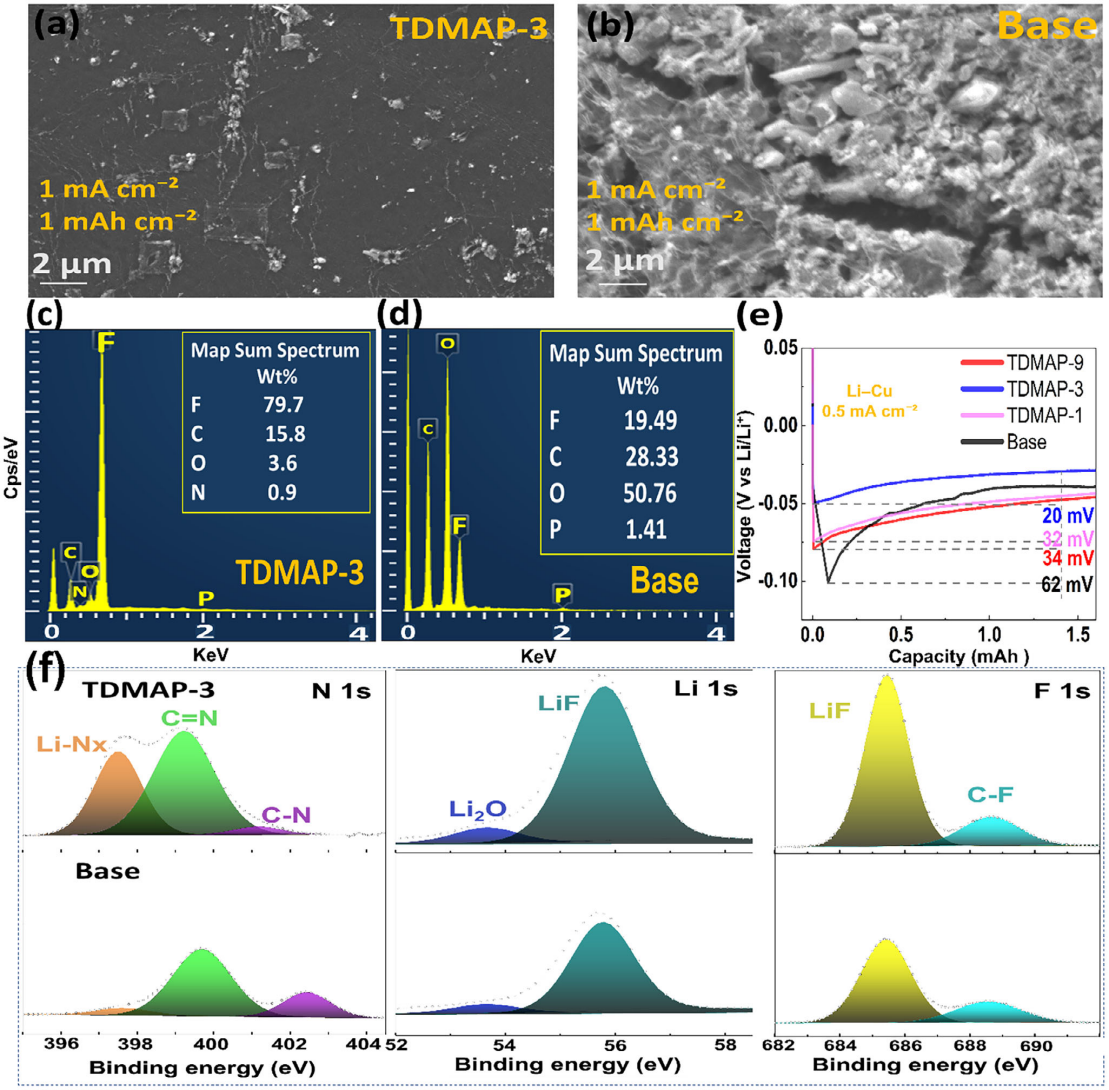
Lithium deposition morphology in the electrolyte of (a) TDMAP‐3 and (b) Base electrolyte in the cycled Li electrodes from Li–Li cells. The corresponding EDS mapping images of C, O, N, F, and P in the (c) TDMAP‐3 and (d) Base electrolyte. (e) Nucleation overpotential curves of the first cycle of Li∥Cu cells at 0.5 mA cm^−^^2^. (f) XPS N 1s, Li 1s, and F 1s spectra of electrodes from the base electrolyte and TDMAP‐3.

After 10 cycles of Li plating/stripping in symmetric Li–Li cells at a current density of 1 mA cm^−^^2^ and a capacity of 1 mAh cm^−^^2^, scanning electron microscope (SEM) images of the Li surface are presented in Figure 3. The Li under TDMAP‐3 (Figure 3a) shows smooth and dendrite‐free Li deposition across the surface, indicating more compact and homogeneous lithium growth, probably due to improved current distribution facilitated by the presence of porphyrin. In contrast, the SEM image of the Li under base electrolyte (Figure 3b) reveals a cracked and porous surface with dendritic structures, reflecting uneven and highly porous lithium deposition. Figure 3c,d show EDS mappings of the cycled Li‐metal anodes in TDMAP‐3 and base electrolytes, respectively. The corresponding EDS analysis indicates that the TDMAP‐3 electrolyte results in a significantly higher and more uniform F signal, about four times greater than that of the base electrolyte, demonstrating the formation of a uniform and conformal SEI. This enhanced signal can be attributed to the increased LiF content in the SEI. The nucleation overpotentials of all electrodes were determined during the first cycle of Li‐Cu cells at 0.5 mA cm^−^^2^, with a capacity of 1 mAh cm^−^^2^ (∼1.77 mAh) (Figure 3e). The porphyrin‐based electrolytes exhibit lower energy barriers for Li nucleation compared to the base electrolyte. Specifically, the nucleation potential for the base electrolyte is the highest at 62 mV, whereas TDMAP‐9, TDMAP‐1, and TDMAP‐3 show progressively lower values of 34, 32, and 20 mV, respectively, with TDMAP‐3 exhibiting the most favorable nucleation behavior.

In order to reveal the reason for the excellent interfacial stability of TDMAPs, X‐ray photoelectron spectroscopy (XPS) was used to analyze the composition of SEI Li‐Li cells after cycling for 10 cycles, as indicated in Figure 3f. The N 1s spectra reveal clear differences between the porphyrin‐containing cell (TDMAP‐3) and the base electrolyte. In TDMAP‐3, three main components are observed at 397.50 eV (Li–N_x_), 399.22 eV (C═N), and 401.15 eV (C─N), whereas in the base electrolyte they appear at 397.65, 399.71, and 402.45 eV, respectively. The uniform downshift of all components in TDMAP‐3 indicates an overall increase in electron density at the porphyrin nitrogen, caused by the electron‐rich tetrakis(4‐N,N‐dimethylaminophenyl)porphyrin. In particular, the more pronounced peak at 397.50 eV supports the formation of lithiated or strongly reduced nitrogen species (Li–N_x_), suggesting that the porphyrin directly coordinates Li^+^ and acts as a Li^+^ reservoir to improve cation distribution at the electrode surface. The C═N component at ∼399 eV corresponds to the porphyrin macrocycle, which provides redox‐active and coordination sites, while the peak contribution at ∼401–402 eV reflects positively charged nitrogen interacting with PF_6_^−^ anions. Together, these features demonstrate that the porphyrin not only undergoes partial lithiation and reduction during cycling, but also stabilizes Li^+^ and PF_6_^−^ at the interface, leading to a more robust and ion‐conductive interphase.

The Li 1s spectra show two main contributions, one at ∼55.7 eV and another at ∼53.6 eV, indicating LiF and Li_2_O, respectively. In both the base and porphyrin‐containing electrolyte, the Li_2_O signal remains relatively minor. However, the porphyrin‐based electrolyte yields a noticeably stronger LiF component, consistent with the F 1s results. This indicates that the electron‐rich porphyrin promotes the preferential conversion of fluorinated electrolyte species into LiF, resulting in a LiF‐rich SEI. Although the porphyrin molecule features an extended π‐conjugated (mesomeric) system that enhances charge delocalization in the electrolyte, the products formed at the interface, primarily LiF, are electronically insulating yet Li^+^‐conductive. Such an interphase is beneficial, as LiF is electronically insulating yet Li^+^‐conductive, thereby suppressing parasitic side reactions and stabilizing Li plating/stripping. The F 1s spectra further highlight the effect of the porphyrin additive. Both electrolytes show two main contributions: LiF at ∼685.4 eV and C─F species at ∼688.6 eV. In the porphyrin‐containing cell (P3), the LiF peak (685.44 eV) is markedly more intense compared to the baseline, indicating that the additive promotes the formation of a LiF‐rich SEI. By contrast, the relative intensity of the C─F component (∼688.65 eV) remains essentially unchanged between the two systems, consistent with its origin from FEC decomposition. Importantly, unlike the N 1s region, no significant binding energy shifts are observed in the F 1s spectra, suggesting that the porphyrin primarily modulates the quantity of LiF formed rather than altering the intrinsic electronic environment of the fluorine species. The enrichment of LiF, a highly stable and ionically conductive SEI component, synergizes with the porphyrin‐mediated Li^+^ coordination (as seen in the N 1s spectra) to reinforce interphase stability and uniform Li deposition as demonstrated by the following electrochemical plating/stripping performances.

**FIGURE 4:**
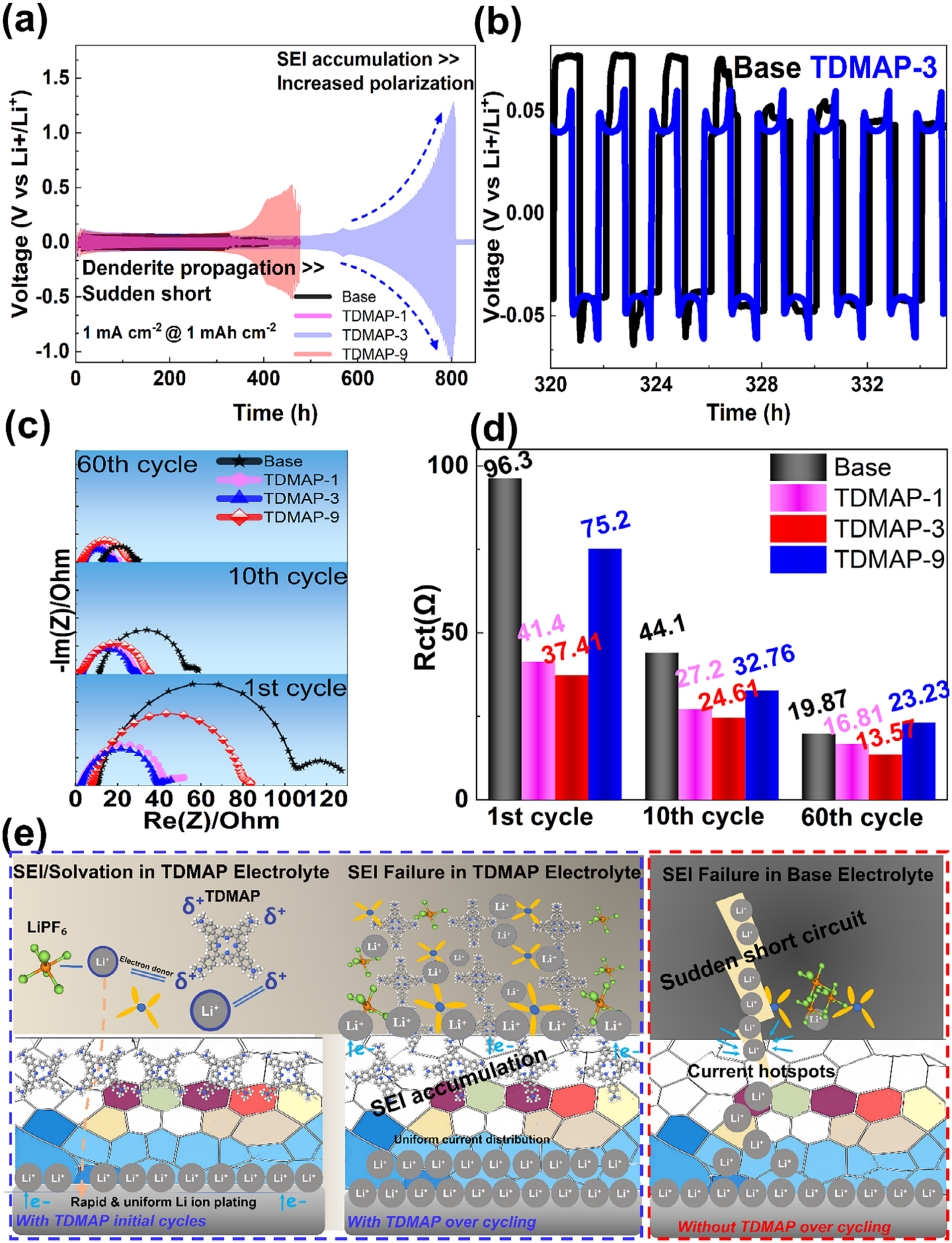
Li plating/stripping and interfacial stability of Li metal cells with TDMAP additives. (a) Voltage profiles at 1 mA cm^−^^2^ for base, TDMAP‐1, TDMAP‐3, and TDMAP‐9 electrolytes. (b) Magnified inset of showing the base‐electrolyte cell after short circuiting. (c) EIS spectra after the first, 10th, and 60th cycles at 25 °C. (d) Extracted charge‐transfer resistance (Rct). (e) Schematic illustration of SEI formation and failure mechanisms, highlighting how TDMAP‐3 promotes uniform SEI and delays dendrite growth.

To evaluate the interfacial stability and compatibility of TDMAP additive, Li plating/stripping tests were conducted at room temperature at 1 mA cm^−^^2^ using Li/TDMAP/Li symmetric cells, along with the base electrolyte for comparison (Figure 4a). After cycling, the base electrolyte and TDMAP‐1 electrolytes exhibited a rapid and continuous decrease in overpotential, ultimately leading to short circuits after approximately 400 and 490 h, respectively, as enlarged in Figure 4b. In contrast, the TDMAP‐3 and TDMAP‐9 cells demonstrated significantly longer lifetimes, lasting for around 500 and 800 h. Notably, the failure mechanisms differed for the higher‐concentration TDMAP electrolytes, showing increased polarization before shorting. This suggests that the SEI formed in the TDMAP‐3 electrolyte is more stable and better compatibility, effectively preventing localized current hotspots and sudden short circuits, as observed in the above SEM and XPS. The SEI promotes uniform potential distribution across the interface, facilitating gradual SEI accumulation over the entire surface and delaying lithium dendrite growth, which ultimately extends the cell's performance [42].

Furthermore, the evolution of interfacial stability was evaluated by electrochemical impedance spectroscopy (EIS) at 25°C after the first, 10th and 60th cycle (Figure 4c). For all electrolytes, the observed polarization trends are consistent with the impedance measurements. The charge‐transfer resistance for all cells decreases over cycling, which can be attributed to the consumption of some electrolyte without reaching complete solvent depletion. Notably, the TDMAP‐3 electrolyte exhibits the lowest charge‐transfer resistance across all cycles, indicating enhanced ion transport and reduced ohmic resistance (Figure 4d). As discussed above, the failure mechanism of the TDMAPs and the base electrolytes is illustrated in Figure 4e.

The electrolyte decomposition behavior was further analyzed by in depth‐profiled XPS. After 10 cycles, the non‐sputtered spectra (0 min) revealed that the TDMAP‐3 electrolyte produces a LiF‐rich surface SEI, whereas the base electrolyte shows comparatively lower LiF intensity and a more organic‐dominated character. Upon extended cycling (60th cycles), depth‐profiled XPS was performed (Figure S4a,b) on both the base and TDMAP‐3 electrolyte at sputtering times of 0, 3, and 10 min. The Li 1s spectra consistently revealed two main contributions, corresponding to LiF and Li_2_O (LiF: 55.7 eV; Li_2_O: 53.6 eV). At all sputtering depths, the electrode with TDMAP‐3 exhibits slightly higher LiF content compared to the base electrolyte. The observed increase in LiF for the base electrolyte at longer cycling arises from different decomposition pathways. With TDMAP‐3, LiF forms rapidly at the SEI surface due to porphyrin‐assisted electrolyte decomposition. In contrast, in the base electrolyte, LiF is generated more gradually through the progressive breakdown of LiPF_6_ /FEC. Consequently, after prolonged cycling, the base electrode “catches up” in LiF content. This trend suggests that the base electrolyte undergoes continuous decomposition, consuming electrolyte species and repeatedly forming a fragile SEI. This behavior is further evidenced by the long‐term cycling of the half‐cells, in which the baseline capacity decays much more quickly.

**FIGURE 5:**
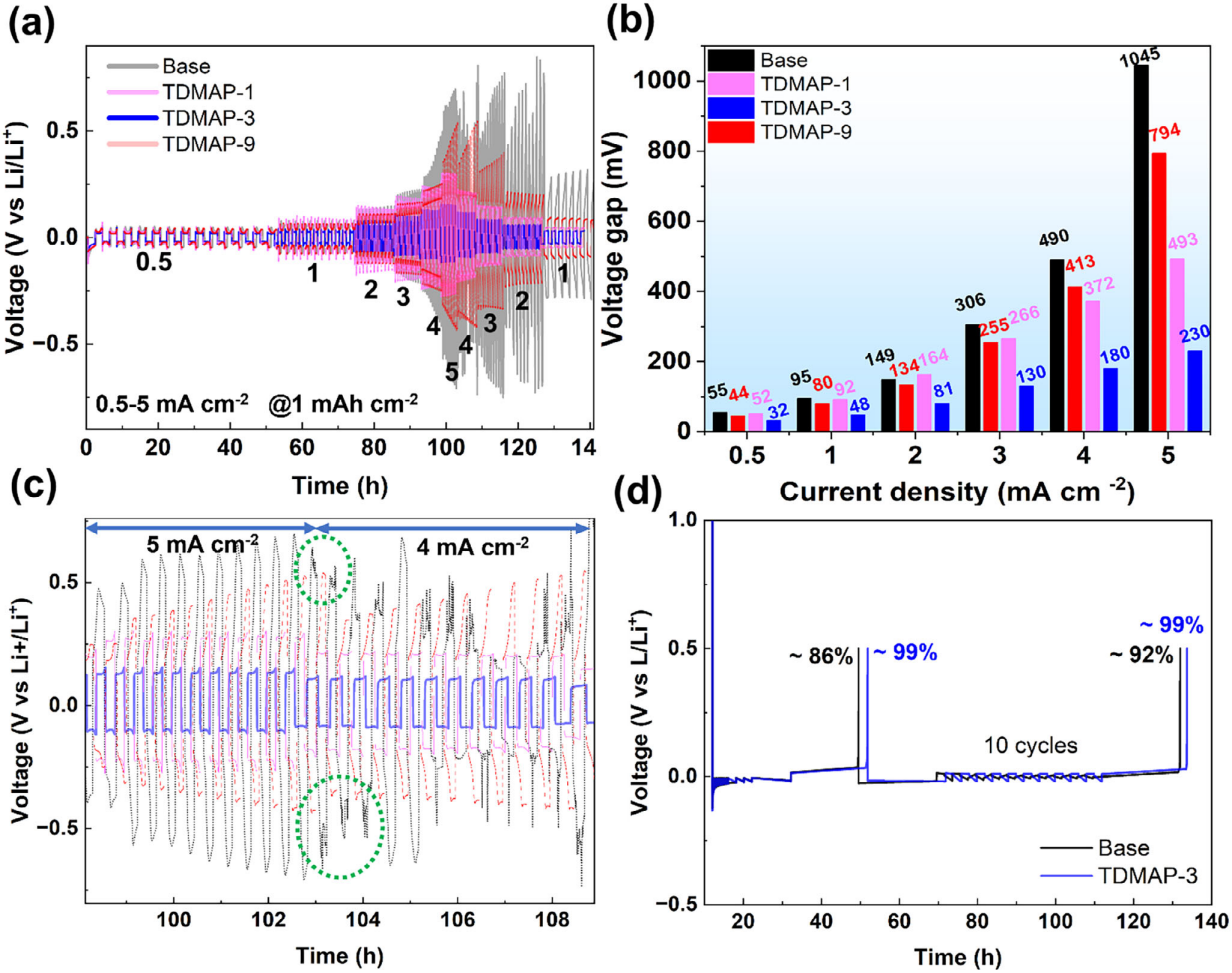
(a) Rate performance of Li–Li symmetric cells with base electrolyte, TDMAP‐1, TDMAP‐3, and TDMAP‐9 at current densities ranging from 0.5 to 5 mA cm^−^^2^. (b) Polarization comparison at various current densities. (c) Magnified inset around 5 mA cm^−^^2^. (d) Average Coulombic efficiency (CE) of Li–Cu asymmetric cells determined using the Auerbach method for base electrolyte and TDMAP‐3.

The stripping/plating behavior of TDMAP‐containing electrolytes on Li metal anodes at various current densities is shown in Figure 5a. At a low current density (0.5 mA cm^−^^2^), no significant difference is observed between the symmetric Li cells with and without the porphyrin additive. As the current density increases, cell polarization rises for all electrolytes, but the base electrolyte exhibits the highest polarization, whereas TDMAP‐3 consistently shows the smallest polarization increase (Figure 5b). At 3 mA cm^−^^2^, pronounced voltage fluctuations appear in the base electrolyte at 5 mA cm^−2^ after approximately 100 h, indicating the onset of partial short circuits (Figure 5c). In contrast, TDMAP‐3 demonstrates excellent rate capability as the current density is increased stepwise from 0.5 to 5 mA cm^−^^2^, with relatively small polarization increases of 48 mV (1 mA cm^−^^2^), 81 mV (2 mA cm^−^^2^), 130 mV (3 mA cm^−^^2^), 180 mV (4 mA cm^−^^2^), and 230 mV (5 mA cm^−^^2^), respectively.

This improved rate performance reflects enhanced interfacial ion transport kinetics, attributed to the reduced Li^+^ desolvation barrier. To minimize the influence of Li losses caused by substrate surface roughness and parasitic reactions between Li and Cu, the Auerbach method was employed to determine the average Coulombic efficiency (CE) during plating/stripping. The CE was measured for Li‐Cu asymmetric cells using the base electrolyte and TDMAP‐3, as shown in Figure 5d. The asymmetric cell employed with TDMAP‐3 achieved an average CE of ∼99%, whereas the base electrolyte exhibited an average CE of ∼92%, indicating the better reversibility of Li plating/stripping behaviors.

**FIGURE 6:**
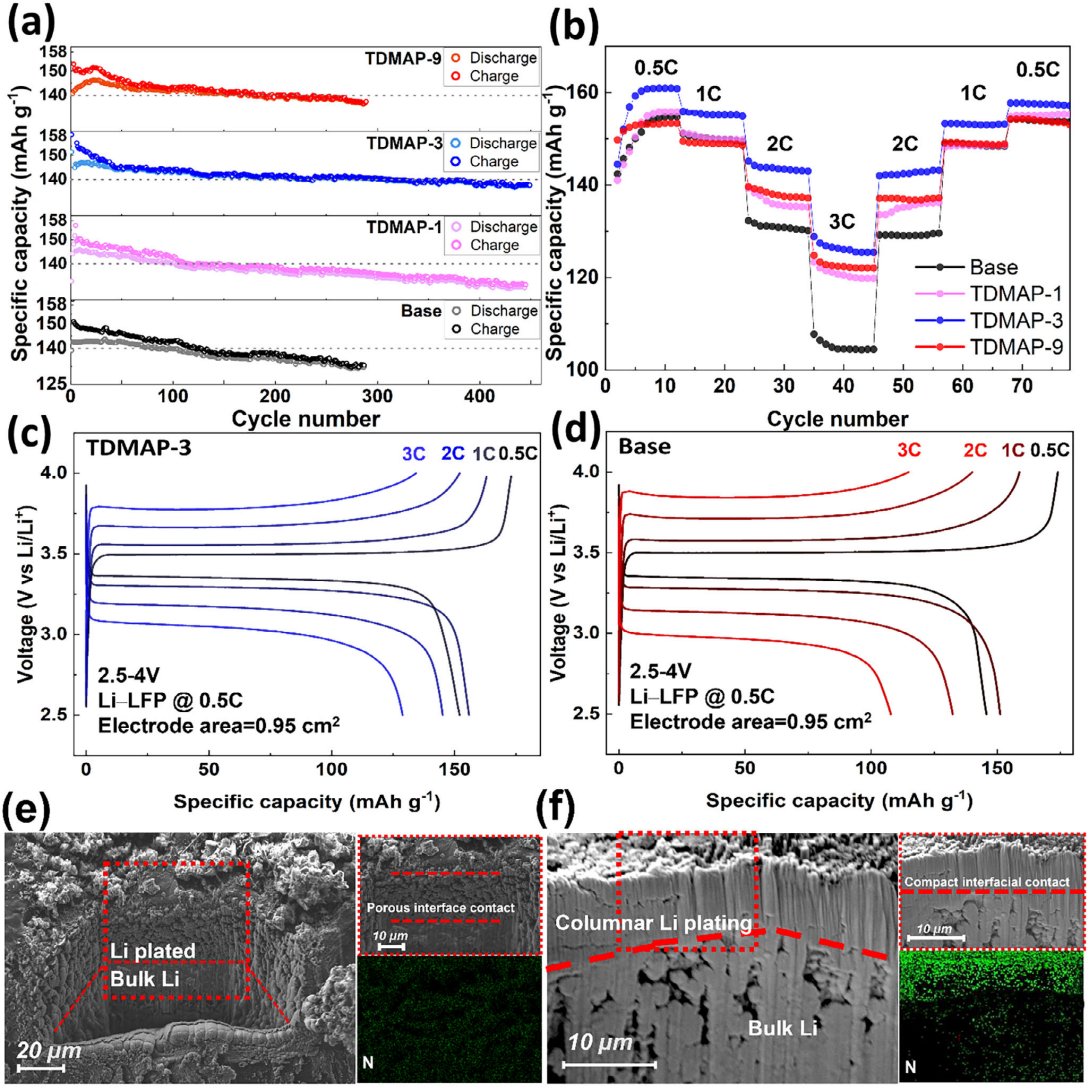
Electrochemical and morphological characterization of Li–LiFePO_4_ full cells with different electrolytes. (a) Cycling performance at 0.5 C (after 4 stabilization cycles at 0.1 C) within 2.5–4 V. TDMAP‐3 exhibits the best long‐term stability (>450 cycles, 99% Coulombic efficiency) (b) Rate performance of full cells, showing highest capacities for TDMAP‐3 (158, 152, 146, and 130 mAh g^−^^1^ at 0.5, 1, 2, and 3 C). Charge–discharge profiles of (c) TDMAP‐3 and (d) base electrolytes at different current densities, highlighting lower polarization in TDMAP‐3. Cryo‐FIB‐SEM cross‐sectional images and corresponding nitrogen (N) elemental maps of Li deposition after rate testing for the Li under (e) base and (f) TDMAP‐3 electrolyte, respectively.

Figure S5 shows the charge‐transfer resistance of the full cells before cycling, where the porphyrin‐based electrolytes exhibited lower charge transfer resistance (Rct) than the base electrolyte. Figure 6a shows the electrochemical performance of Li‐LiFePO_4_ full cells with different electrolytes. Cells were stabilized for 4 cycles at 0.1 C, then cycled at 0.5 C within 2.5–4 V. TDMAP‐3 and TDMAP‐9 exhibited the highest initial discharge capacities of 144.8 and 151.5 mAh g^−^^1^, outperforming base (142.5) and TDMAP‐1 (142.6). The cell with base electrolyte had the largest voltage gap, while the curves in the cell with TDMAP‐3 nearly overlapped, indicating superior reversibility and capacity retention. Using 140 mAh g^−^^1^ as a threshold, the cell with base electrolyte only lasted 73 cycles above it and failed after 287 cycles, with an average Coulombic efficiency of 98.6% and unstable cycling. TDMAP‐9 lasted 289 cycles, TDMAP‐1 lasted ∼445 cycles, and TDMAP‐3 maintained stable cycling over 450 cycles. Notably, the cell with TDMAP‐3 exhibited a stable Coulombic efficiency of 99% throughout cycling (Figure S6). After 280 cycles, discharge capacities were 136, 141, 136, and 132 mAh g^−^^1^ for TDMAP‐1, TDMAP‐3, TDMAP‐9, and base electrolyte, corresponding to retentions of 89.7%, 97.3%, 95.3%, and 92.6%, respectively. At the 400th cycle, TDMAP‐1 and TDMAP‐3 had 131.5 and 139 mAh g^−^^1^ (86.8% and 95.9% retention). TDMAP‐3 thus demonstrated the best one in cycling stability, capacity retention, and SEI stability.

The rate capability of Li‐LFP full cells with different electrolytes is presented in Figure 6b. TDMAP‐3 delivered the highest capacities, reaching 158 mAh g^−^^1^ at 0.5 C after two stabilization cycles, and 152, 146, and 130 mAh g^−^^1^ at 1 C, 2 C, and 3 C, respectively. A clear trend was observed, with capacities following the order: TDMAP‐3> TDMAP‐9> TDMAP‐1> Base. The cell with base electrolyte exhibited a pronounced capacity loss at 3 C, evidencing sluggish reaction kinetics. Upon returning the current to 0.5 C, the TDMAP‐based cells nearly recovered their initial capacities, confirming the improved ion transport and smoother SEI formation provided by the additives. Charge–discharge profiles of TDMAP‐3 and base electrolyte at various current densities are shown in Figure 6c,d. In addition, Figure S7 highlights the polarization gap, where the cell with base electrolyte consistently displayed higher polarization than the TDMAP‐based electrolytes at all current densities. Although excess Li is used in the LiFePO_4_||Li cell, the improved cycling performance with TDMAP‐3 is attributed to the formation of a more stable, LiF‐rich interphase at the anode, which suppresses parasitic reactions and enhances long‐term stability.

To investigate lithium morphology on the anode, cryo‐FIB‐SEM was performed on the Li metal side of Li‐LiFePO_4_ full cells, including base electrolyte and TDMAP‐3 electrolyte after rate performance testing (Figure 6e,f, respectively). The cross‐sectional images show that Li exhibited dendritic growth in the base electrolyte (Figure 6e), and a large network of a porous interfacial contact and significant voids are formed at the Li bulk‐Li plated interface. In contrast, Li deposited from TDMAP‐3 (Figure 6f) formed significantly denser and more compact deposits. The preferred columnar Li growth with conformal Li‐Li interfacial contact observed for TDMAP‐3 can be attributed to the additive promoting preferential Li deposition. The EDS nitrogen mappings of both surfaces are also shown, indicating higher N content on the TDMAP‐3 surface due to the nitrogen in the TDMAP additive. The cross‐sectional elemental compositions are also provided in Figure S8. Additionally, surface SEM images of the same electrodes were obtained (Figure S9). The Li electrode coupled with the base electrolyte exhibits porous Li plating, as confirmed by the corresponding EDX elemental analysis, whereas the one with TDMAP‐3 shows higher F and N contents and maintains smooth and uniform Li deposition even after prolonged high‐rate cycling.

## 3 Conclusions

In summary, tetrakis(4‐N,N‐dimethylaminophenyl) additive, particularly TDMAP‐3, effectively modulates Li^+^ solvation by displacing EC molecules from the first solvation shell and creating partially mixed coordination environments (Li^+^–NMe_2_ and Li^+^–PF_6_^−^), as evidenced by FTIR and Raman spectroscopies. SEM and EDX analyses revealed dendrite‐free Li deposition and a LiF‐rich and nitrogen‐rich SEI in TDMAP‐3, in contrast to porous Li formed in the base electrolyte. Nucleation potentials were reduced from 62 mV in the base electrolyte to 20 mV in TDMAP‐3, indicating more favorable Li nucleation. XPS showed that Li^+^ partially coordinates with the porphyrin macrocycle, allowing it to act as a Li^+^ reservoir that improves cation distribution and promotes LiF‐rich SEI formation. The Li‐LiFePO_4_ full cell with TDMAP‐3 delivered capacities up to 158 mAh g^−^^1^ at 0.5 C, maintained retention of 97.3% after 280 cycles, and exhibited a Coulombic efficiency of ∼99%. Overall, TDMAP‐3 optimizes Li^+^ solvation and distribution, promotes a stable, ionically conductive SEI, and enables uniform, long‐lasting Li plating/stripping even at high current densities, whereas too low (TDMAP‐1) or too high (TDMAP‐9) concentrations result in suboptimal deposition behavior and aggregation, showing the potential for practical applications.

## Author Contributions

**Pooria Afzali** performed the electrochemical experiments, analyzed the data, and wrote the original manuscript. **Sergio Rodriguez** and **Jin‐Hyun Chang** performed the simulation. **Jian Wang** and **Maximilian Fichtner** supervised the work, provided guidance on data interpretation, and reviewed and edited the manuscript. **Luca Magagnin** reviewed the manuscript. All authors discussed the results and approved the final version of the manuscript.

## Conflicts of Interest

The authors declare no conflicts of interest.

## Supporting information




**Supporting file**: advs76009‐sup‐0001‐SuppMat.docx

## Data Availability

The data that support the findings of this study are available from the corresponding author upon reasonable request.
