# Supplementary material for: Concentration‐Driven Li+ Solvation Engineering with TDMAP‐Based Porphyrin Additives for Dendrite‐Free Li Metal Batteries
Source: Adv Sci (Weinh). 2026 Jun 9:e76009. Online ahead of print. doi: 10.1002/advs.76009 (PMC13337111; doi:10.1002/advs.76009)
Supplement: Supplementary file 1 — Supporting file: advs76009‐sup‐0001‐SuppMat.docx [file ADVS-9999-e76009-s001.docx]

**Concentration-Driven Li⁺ Solvation Engineering with TDMAP-Based Porphyrin Additives for Dendrite-Free Li Metal Batteries**

*Pooria Afzali ^abc^, Jian Wang^ac*^, Sergio Rodriguez^e^, Jin-Hyun Chang^e^, Luca Magagnin ^b*^, Maximilian Fichtner^acd^**

^a^ Helmholtz Institute Ulm for Electrochemical Energy Storage (HIU), Helmholtzstr. 11, 89081 Ulm, Germany

^b^ Dipartimento di Chimica, Materiali e Ingegneria Chimica “Giulio Natta”, Politecnico di Milano Via Luigi Mancinelli 7, Milan 20131, Italy

^c^ Institute of Nanotechnology, Karlsruhe Institute of Technology (KIT), Karlsruhe D76021, Germany

^d^ Ulm University, Institute for Inorganic Chemistry II, Albert-Einstein-Allee 11, 89081 Ulm, Germany

^e^  Department of Energy Conversion and Storage, Technical University of Denmark, DK-2800 Kgs, Lyngby, Denmark

**Corresponding authors**: jian.wang@kit.edu; luca.magagnin@polimi.it, m.fichtner@kit.edu

**1. Experimental section**

1.1 Materials preparation and cell assembly

All electrolyte solutions were prepared in an argon-filled glovebox (H₂O and O₂ < 0.1 ppm). Tetrakis(4-N,N-dimethylaminophenyl)porphyrin (TDMAP, Sigma-Aldrich) was first dried at 110 °C in an oven before being transferred into the glovebox. The base electrolyte was then prepared by dissolving lithium hexafluorophosphate (LiPF₆, 1 M) in a 1:1 (v:v) mixture of ethylene carbonate (EC) and dimethyl carbonate (DMC), containing 10 wt% fluoroethylene carbonate (FEC). TDMAP was added to this base electrolyte to yield final concentrations of 1, 3, and 9 mg mL^-1^. Each mixture was stirred at 300 rpm for 12 h at room temperature to ensure complete dissolution of TDMAP. These electrolytes were subsequently used for assembling Li–LiFePO_4_ full cells, Li–Li symmetric cells and Li-Cu asymmetric cell.

The cathode slurry was prepared by mixing commercial lithium iron phosphate (LiFePO₄) as the active material, Super P carbon as the conductive additive, and polyvinylidene fluoride (PVDF) binder in an 8:1:1 weight ratio in N-methyl-2-pyrrolidone (NMP) solvent. The slurry was then uniformly coated onto aluminum foil current collectors. After coating, the electrodes were dried under vacuum at 90 °C for 12 h. The dried LiFePO₄ cathodes had an average active material loading of approximately 3–4 mg cm⁻².

Symmetric Li–Li cells were prepared in an Ar-filled glovebox (H₂O, O₂ < 0.1 ppm) using CR2032-type coin cells, incorporating two lithium foil electrodes (15.6 mm diameter) separated by a glass fiber separator. Full Li–LiFePO_4_ cells were assembled with a lithium foil anode (15.6 mm diameter) and an LFP cathode (11 mm diameter). In both cell types, 40 µL of the previously prepared electrolyte was added to each cell before crimp sealing.

1.2 Material Characterizations

Raman measurements were carried out for different electrolytes with a confocal Raman microscope (InVia, Renishaw) in the spectral range of 200–2000 cm^–1^ using a 532 nm laser excitation. The [average laser power](https://www.sciencedirect.com/topics/engineering/average-laser-power) was kept at ∼1 mW and exposure times varied from 20 s to 120 s depending on sample response. Also, Fourier-transform infrared (FTIR) spectra were recorded on a PerkinElmer Spectrum Two spectrometer for the same electrolytes

SEM, EDX, and focused ion beam (FIB) cross-sectional analyses of cycled electrodes were performed using a Crossbeam XB340 (ZEISS) field-emission scanning electron microscope equipped with a Ga-ion source (Capella, ZEISS) and an integrated energy-dispersive X-ray spectrometer. Imaging was carried out at a working distance of ~5 mm using a secondary electron detector. Cross-sections were prepared by FIB milling (30 nA) followed by polishing (3 nA). All specimens were prepared in an Ar-filled glovebox and transferred into the instrument using a transfer shuttle box to prevent air exposure.

Additionally, high-resolution X-ray photoelectron spectroscopy (XPS) was conducted to analyze the chemical composition of the SEI layer on a Specs XPS system with a Phoibos 150 energy analyzer. To avoid surface contamination, the samples were transferred in inert gas atmosphere to the sample load lock of the XPS system. All binding energies were calibrated to the C1s peak of C species (at 284.8 eV). For some samples a depth profile of elemental concentrations was collected by removal of the topmost surface layers with successive sputtering for 3 and 7 minutes, resulting in total sputter times of 0, 3, and 10 minutes. The peak fitting of the XPS results was done with CasaXPS, using Shirley-type backgrounds and Gaussian-Lorentzian (GL30) peak shapes.

1.3 Electrochemical Measurements

Electrochemical stability window (ESW) of the electrolytes was determined through linear sweep voltammetry (LSV) using Li/TDMP-3/SS and Li/Base electrolyte/SS coin cell configurations, with a sweep rate of 1 mV s^−1^. To test the ionic conductivity of liquid TDMAPs and base electrolytes, the cells were assembled in CR2032-type coin cells rested for 10 h before testing. The impedance spectra of symmetric Li–Li electrodes were collected from 100 mHZ to 200 kHz using VMP-3 Instruments (BioLogic). Based on the **Equation** 1, the ionic conductivity (*σ*) can be calculated, in which *d* is the impacted thickness of separator (cm), S represents the area of the effective area (cm^2^), and *R* is the intrinsic impedance (Ω), tested by electrochemical impedance spectroscopy (EIS).

$\sigma=\frac{d}{R\times S}$ (1)

A series of *σ* in temperature range of 20-80 °C were tested and calculated. According to the Arrhenius equation (2), the active energy (*E_a_*) could be calculated to well reveal the kinetic of these electrolyte systems:

$\sigma=A\exp(-E_{a}/RT)$ (2)

where *E_a_*, *R*, *T*, *A*, *σ* are active energy, Boltzmann constant, absolute temperature, pre-exponential factor, ionic conductivity at different temperatures.

The Li^+^ transference number (t_Li_^+^) can be calculated by Bruce-Vincent method:

$t_{Li^{+}}=\frac{I_{SS}(\Delta V-I_{0}R_{0})}{I_{0}(\Delta V-I_{SS}R_{SS})}$ (3)

where ΔV is the applied constant potential (10 mV) during the polarization process, I_0_ and I_ss_ are the initial and steady-state currents, R_0_ and R_ss_ are the initial and steady-state resistance values, respectively.

1.4 Simulation method

Density-functional theory (DFT) calculations were carried out with Gaussian 16. Gas-phase geometries of TDMAP, ethylene carbonate (EC), and their Li⁺ complexes were optimized with B3LYP and the 6-311G(d,p) basis set. Each stationary point was checked by a frequency calculation at the same level; no imaginary modes were found, so the structures were treated as local minima.

Electronic energies were refined, and frontier orbitals were evaluated, with single-point calculations in gas phase on those geometries using ωB97XD with def2-TZVP. ωB97XD was used because it tends to treat dispersion and long-range effects reasonably well when comparing Li⁺ binding energies.

Li⁺–ligand binding energies were defined as

ΔE_binding energy_ = E_substrate+Li_ − (E_substrate_ + E_Li_)

where E_substrate+Li_, E_substrate_, and E_Li_ are the electronic energies of the bonded complex (Li + molecule), the free molecule, and Li, respectively. HOMO and LUMO energies from the ωB97XD/def2-TZVP single points were used as a simple readout of nucleophilicity and reduction stability.

**2. Supporting Figures**

**
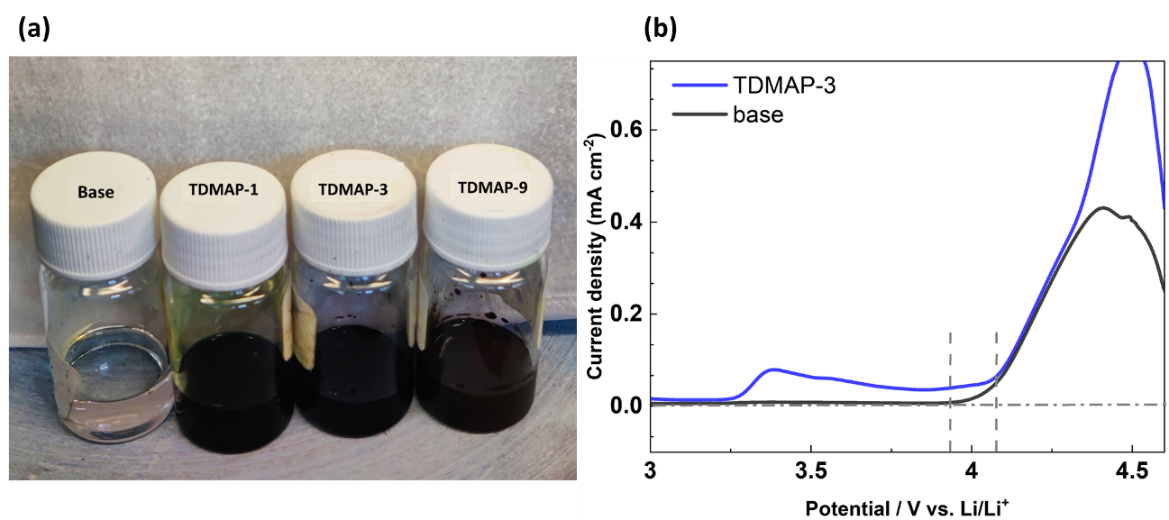
**

Figure S1. (a) Optical image of precursor solutions. (b) Cathodic LSV for the Base electrolyte and TSMAP-3. Scan rate: 1 mV s^−1^


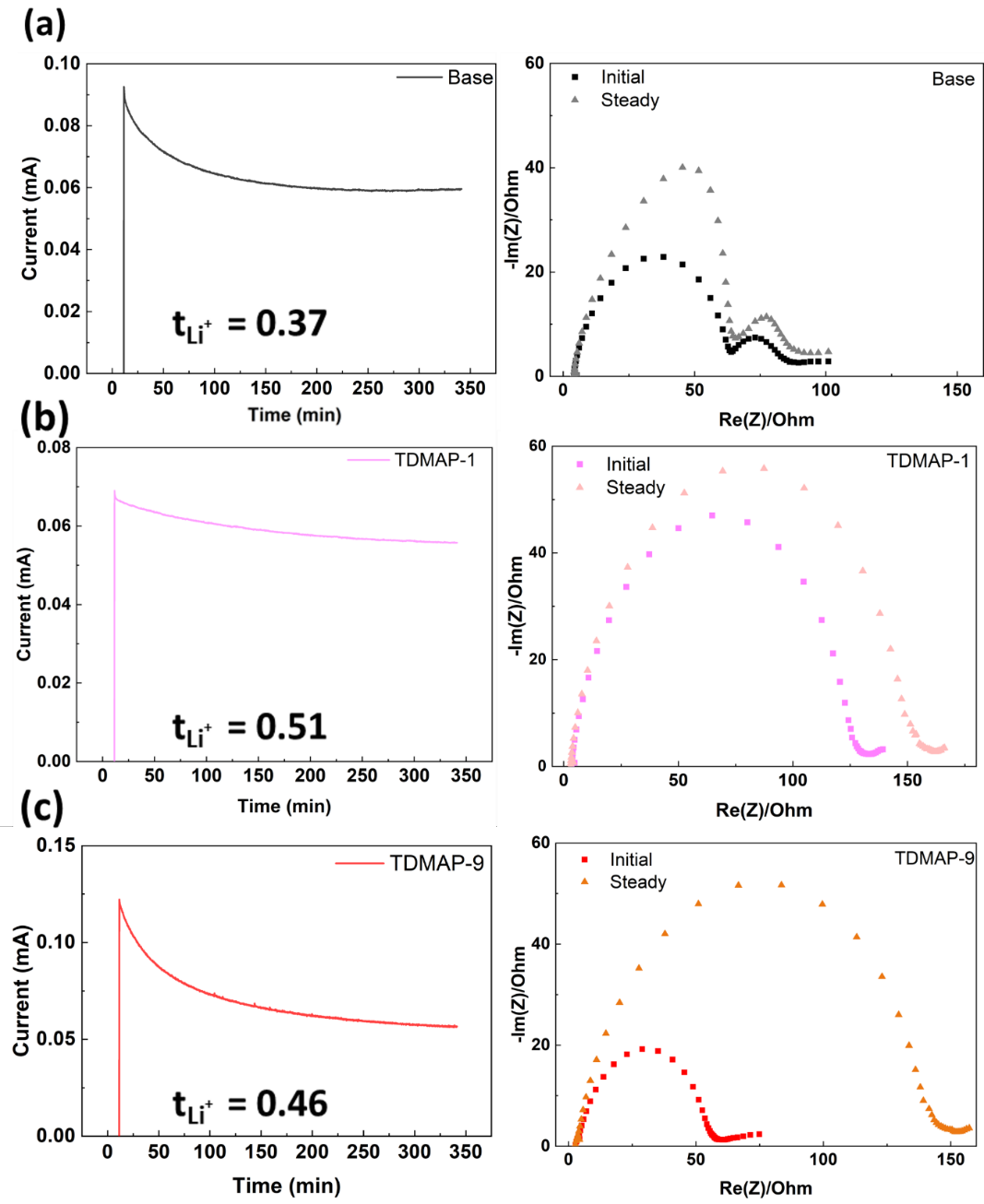


Figure S2. Chronoamperometry (CA) and Nyquist plots of Li–Li symmetric cells using (a) Base, (b) TDMAP-1 and (c) TDMAP-9 electrolytes to calculate Li^+^ transference number.


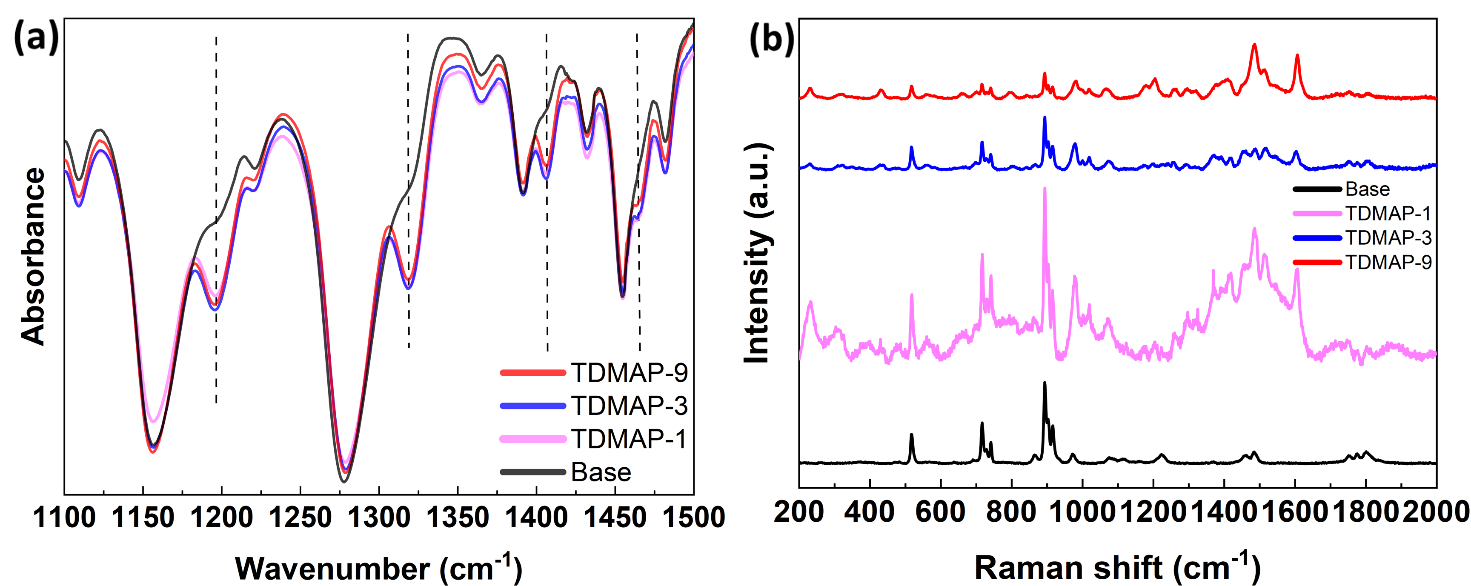


Figure S3. (a) Full FT-IR spectra of as prepared electrolytes at 1100−1500 cm^−1^; (b) Full Raman spectra of as prepared electrolytes at 200 – 2000 cm^−1^ illustrating the shifts and molecular interactions.


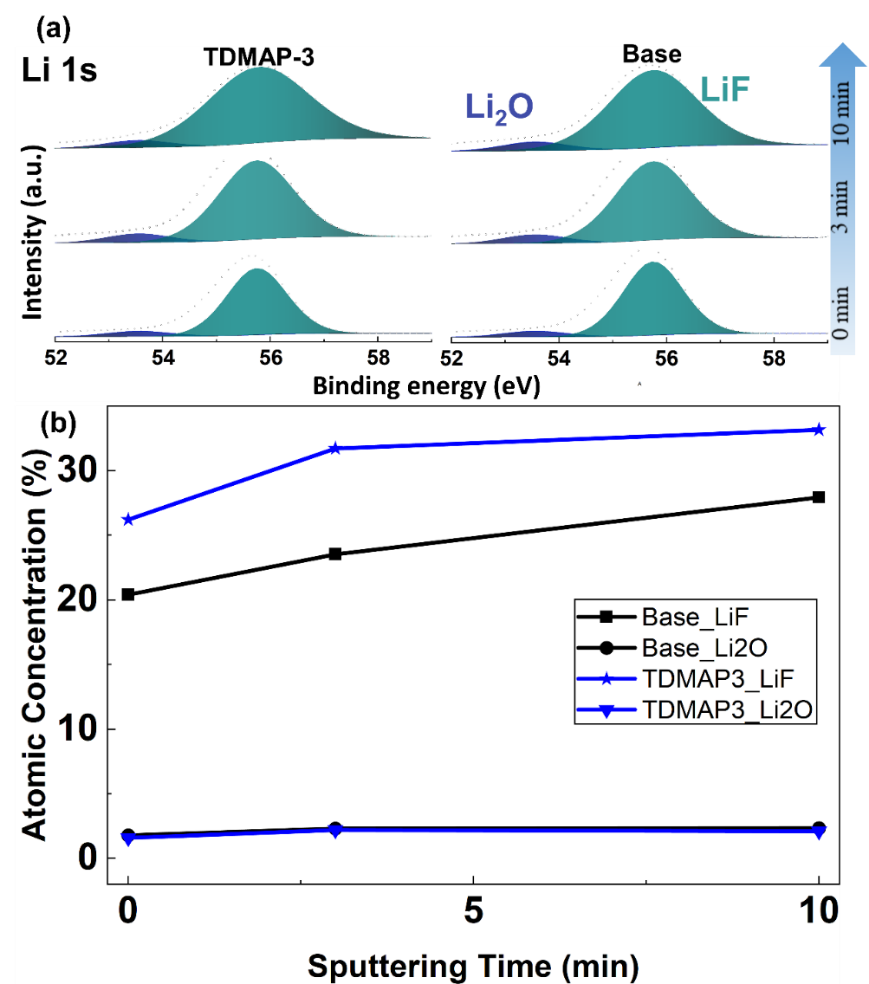


Figure S4. XPS analysis of Li electrodes after 60 cycles. (a) Li 1s spectra at sputtering times of 0, 3, and 10 min for base and TDMAP-3 electrolytes. (b) Concentration of Depth-profile at 0, 3, 10 min.

**
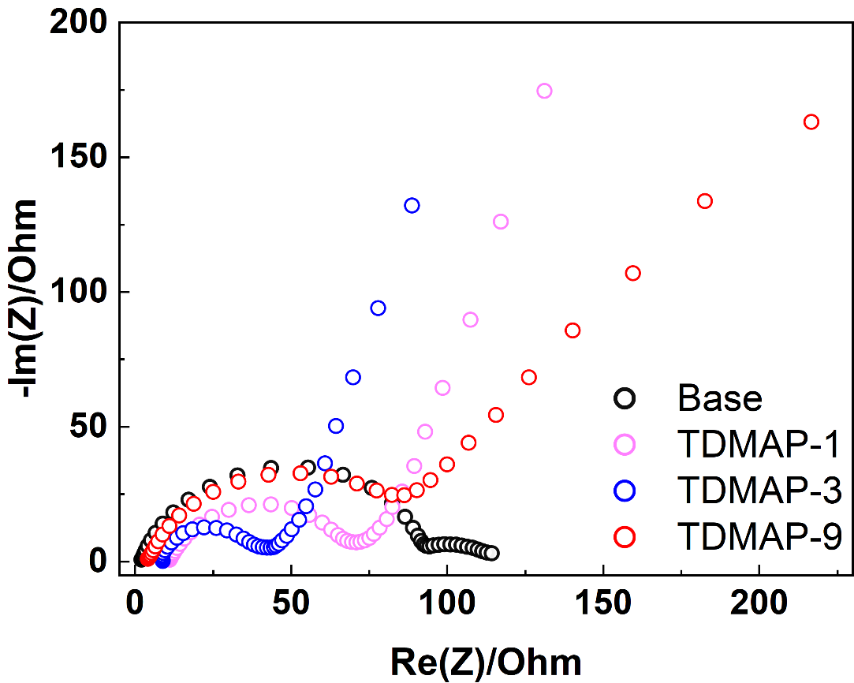
**

Figure S5. Charge-transfer resistance (R_ct_) of Li–LiFePO_4_ full cells before cycling.

**
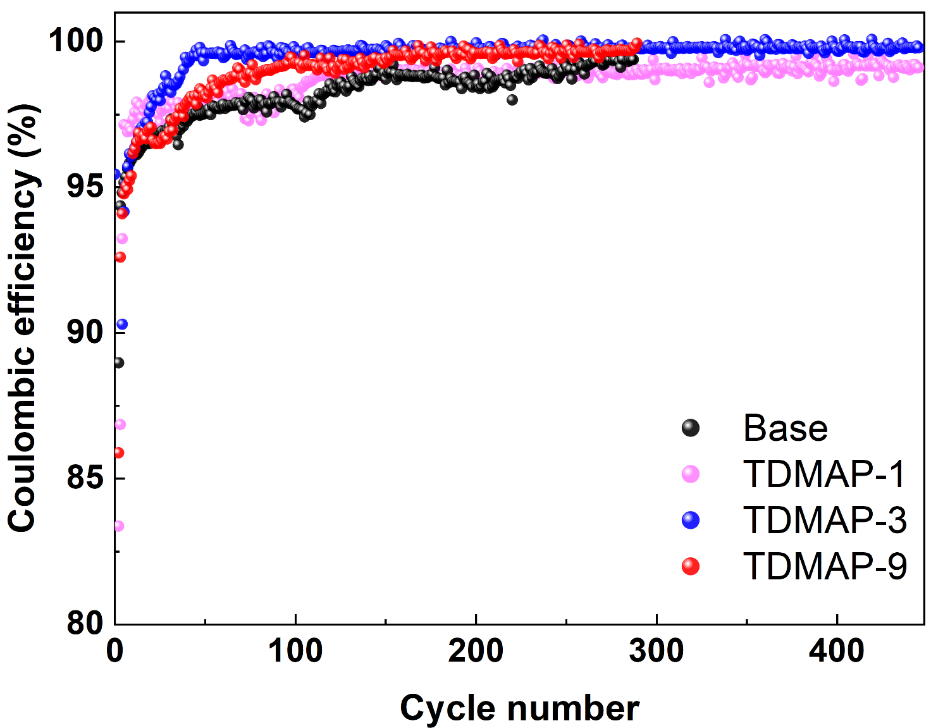
**

Figure S6. Coulombic efficiency (CE) of Li–LiFePO_4_ full cells with different electrolytes during long-term cycling.


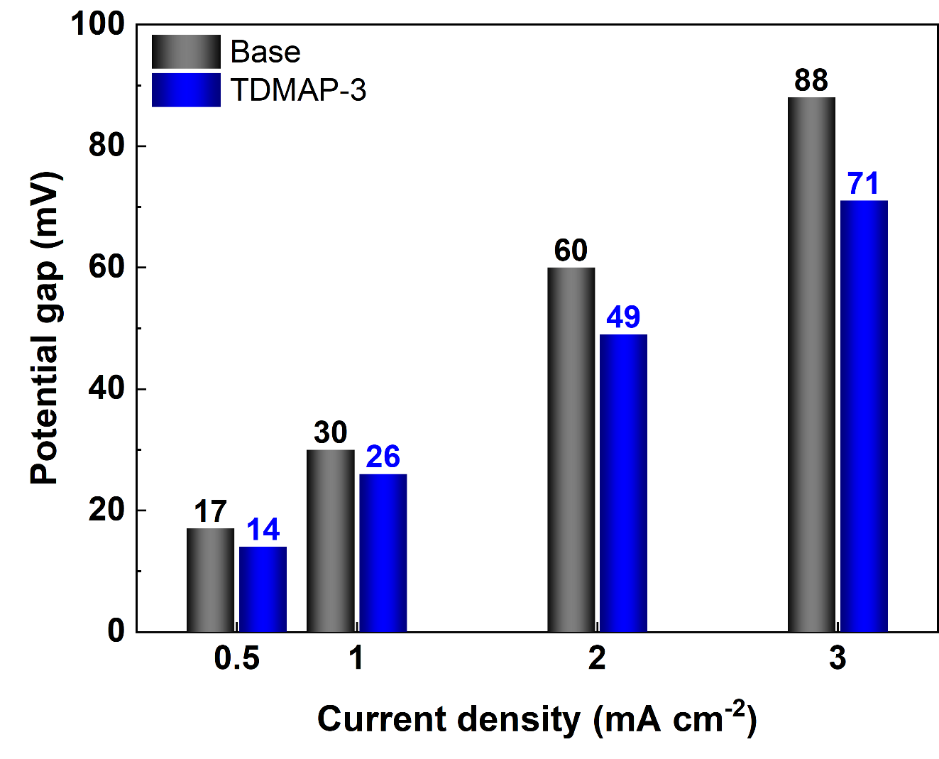


Figure S7. Polarization gap between charge and discharge plateaus of Li–LiFePO_4_ full cells with TDMAP and base electrolytes.


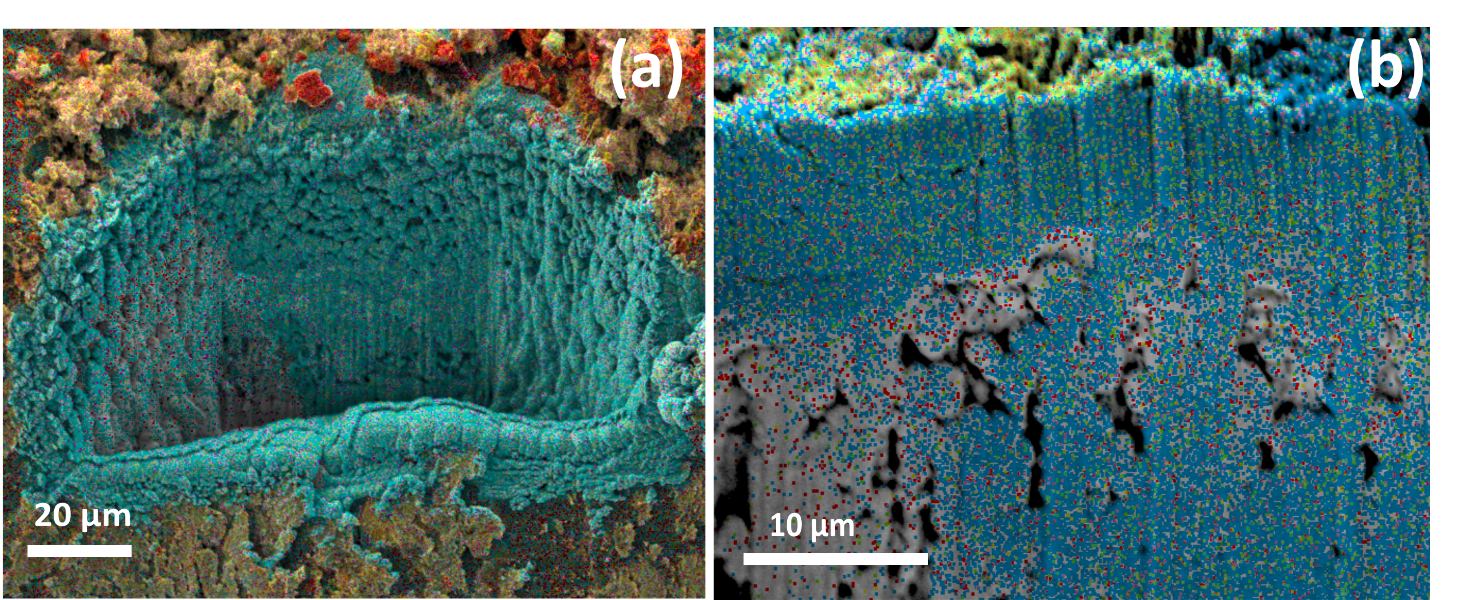


Figure S8. EDX cross-section color maps of Li metal electrodes: (a) Base electrolyte and (b) TDMAP-3 electrolyte.


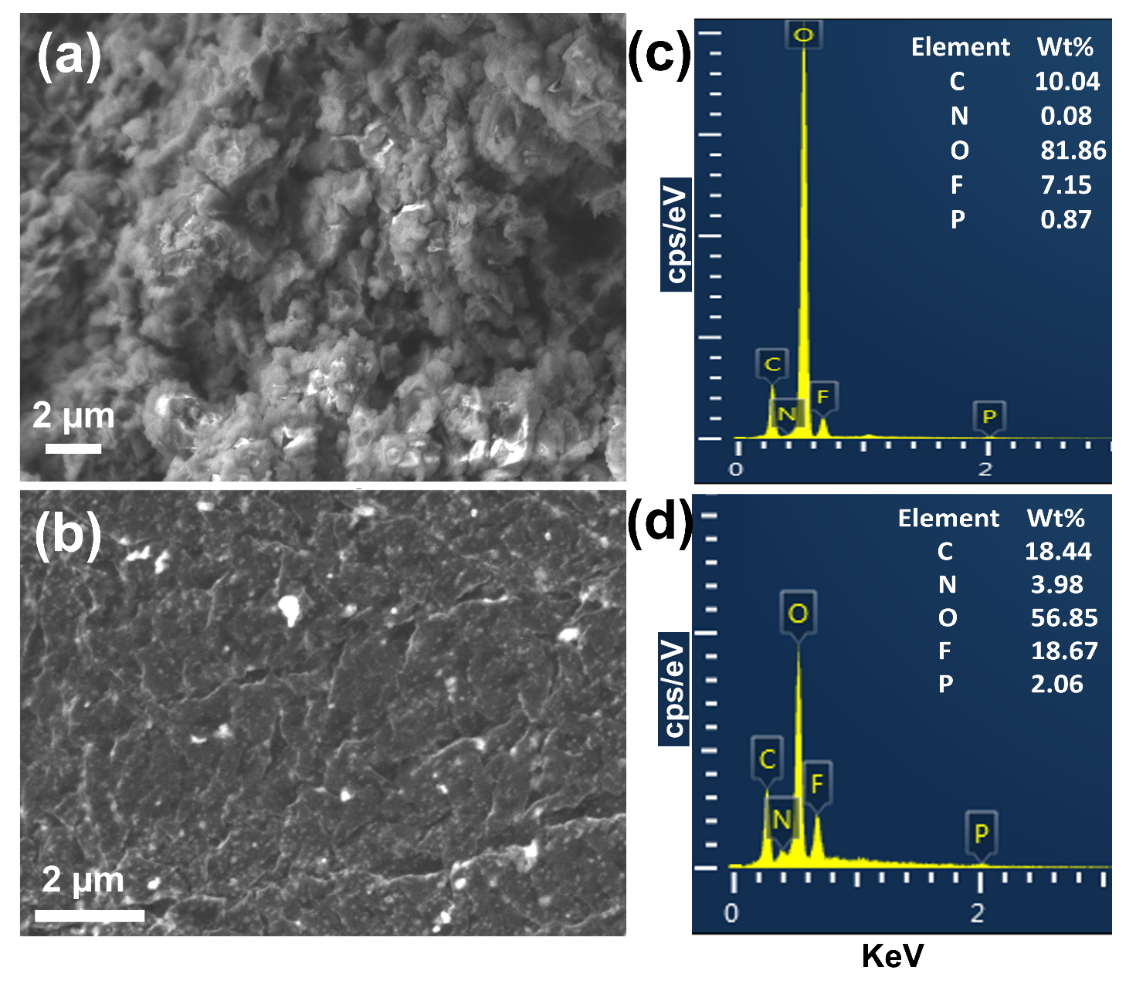


Figure S9. Surface characterizations of Li metal electrodes after electrochemical rate performance of full cells: SEM image of Li plated from the (a) base electrolyte and (b) TDMAP-3, corresponding EDX elemental compositions of (c) base electrolyte and (d) TDMAP-3.
